# Non-thermal atmospheric pressure plasma as a powerful tool for the synthesis of rhenium-based nanostructures for the catalytic hydrogenation of 4-nitrophenol

**DOI:** 10.1039/d1ra07416d

**Published:** 2021-12-01

**Authors:** Piotr Cyganowski, Dominik Terefinko, Piotr Jamroz, Pawel Pohl, Anna Dzimitrowicz

**Affiliations:** a Department of Process Engineering and Technology of Polymer and Carbon Materials, Faculty of Chemistry, Wroclaw University of Science and Technology Wybrzeze Stanislawa Wyspianskiego 27 50-370 Wroclaw Poland piotr.cyganowski@pwr.edu.pl; b Department of Analytical Chemistry and Chemical Metallurgy, Faculty of Chemistry, Wroclaw University of Science and Technology Wybrzeze Stanislawa Wyspianskiego 27 50-370 Wroclaw Poland

## Abstract

Here we have presented a new method for the synthesis of Re nanostructures with defined optical, structural, and catalytic properties. The Re-based nanoparticles (NPs) were obtained using a reaction-discharge system that is unique in its class, because of its working in the high-throughput mode. Within this application, direct current atmospheric pressure glow discharge (dc-APGD) was used as a non-thermal atmospheric pressure plasma (NTAP) source, which led to the reduction of Re(vii) ions and the formation of Re nanostructures through the plasma–liquid interactions. The Re-based NPs were synthesized in a flow-mode reaction-discharge system, where their precursor solution was a flowing liquid anode (FLA) or a flowing liquid cathode (FLC). The resultant NPs were analyzed using UV/Vis absorption spectrophotometry and transmission electron microscopy (TEM), which were supported by selected area X-ray diffraction (SAED) and the energy dispersive X-ray spectroscopy (EDX). Additionally, the mechanism for the reduction of Re(vii) ions was explained by the differences in the concentrations of the selected reactive nitrogen species (RNS) and reactive oxygen species (ROS) produced by dc-APGD. It was found that the application of dc-APGD, operating in a FLA configuration (FLA-dc-APGD), resulted in the formation of ReNPs with Re^0^, while the use of dc-APGD operating in a FLC configuration (FLC-dc-APGD) led to the formation of Re oxide NPs. In the latter case, a much greater oxidizing environment was likely provided, therefore the RNS and ROS contributed to the formation of Re oxide nanostructures. The ReNPs with Re^0^ were characterized by a size of 6.02 ± 3.01 nm, and the Re oxide NPs were characterized by a size of 4.97 ± 3.82 nm. Both types of nanostructures were then employed in the catalytic hydrogenation of 4-nitrophenol (4-NP) to 4-aminophenol (4-AP). Based on the results, both of the nanocatalysts effectively reduced 4-NP with an apparent rate constant (*k*_app_) of 2.6 × 10^−3^ s^−1^. At the same time, the catalytic activity was linked with the average size distribution of the Re nanostructures, as opposed to their morphology.

## Abstract 2

A unique reaction-discharge system was used for the production of Re-based nanoparticles (NPs) revealing enhanced catalytic activity.

## Graphical abstract

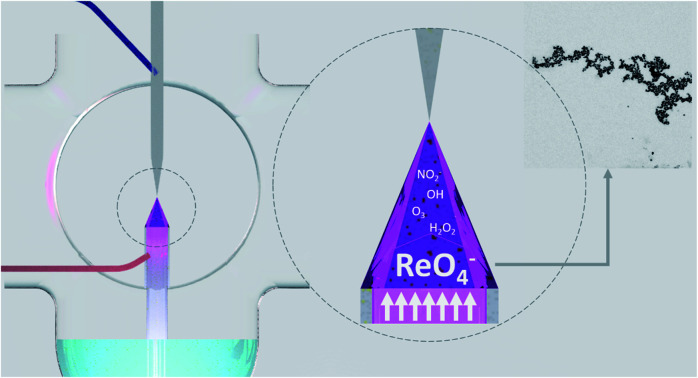

## 1. Introduction

In recent years, the expeditious development of non-thermal atmospheric pressure plasma (NTAP) sources for defined applications, including wound healing,^1,2^ the eradication of bacteria,^3,4^ seed germination,^5^ wastewater purification,^6^ and cancer cell apoptosis^7^ has been observed. Apart from the above-mentioned examples of NTAP applications, its most leading use is related to the synthesis of nanomaterials, including gold nanoparticles (AuNPs),^8–12^ silver nanoparticles (AgNPs),^9,13–16^ platinum nanoparticles (PtNPs),^17^ zinc oxide nanoparticles (ZnONPs),^18^ core–shell nanostructures,^19^ and magnetic nanoparticles (MagNPs).^12^ It is well known that during NTAP operation in reaction-discharge systems developed for the above purposes, selected reactive oxygen species (ROS) and reactive nitrogen species (RNS) are generated. The so-produced ROS and RNS exhibit a defined red-ox potential towards the reduction of metals ions to their proper metallic form of nanometric size.^20^ Additionally, the possibility of tailoring the operating parameters of NTAPs allows metallic nanostructures of certain characteristics to be obtained, which in turn defines their subsequent applications.

Among the nanostructures utilized in catalysis, rhenium nanoparticles (ReNPs) are one of the most tempting alternatives to the currently used solutions.^21–23^ Re-based materials are of direct interest to the chemical and petrochemical industries, and serve as a highly efficient catalyst for the production of gasolines with a high-octane number, ammonia gas, and hydrocarbons.^24,25^ ReNPs offer an increased surface area of Re-based catalysts, and as such they are recognized as the most promising nanomaterials. The research on the synthesis of ReNPs is particularly tempting. According to a few literature reports, this unique type of nanomaterials has already outperformed other NP-based catalysts with regards to the graphitization of phenyl groups,^26^ the transformation of olefins,^21^ and the reduction of nitroaromatic compounds.^23,27^ However, the literature lacks information on the synthesis and applications of ReNPs in contrast to the other nanostructures for which the synthesis protocols are described in *e.g.* ref. 28–30. In a few reports, ReNPs were obtained using advanced high-energy output physical methods, including the deposition of Re vapours using pulsed-lasers, high frequency electric current and ionizing radiation.^26,31,32^

To the best of our knowledge, there are no works in which the application of NTAPs for the synthesis of ReNPs has been studied. In this context, the application of direct current atmospheric glow discharge (dc-APGD), as the NTAPs source, for the production of ReNPs could be a much easier and cheaper alternative to the physical methods that have already been proposed. When compared to other methods, the application of dc-APGD for the synthesis of ReNPs provides a number of advantages. Firstly, the ignition of dc-APGD is relatively easy and does not require any extra safety precautions.^9^ Secondly, it generates several ROS and RNS, which, along with hydrated electrons (e_aq_^−^), are involved in the reduction of the ReNPs precursor without the addition of other reducing agents. Thirdly, dc-APGD provides electrostatic stabilization of the resulting ReNPs, and enables the application of capping agents to be omitted.^8^ Fourth, because there is a possibility for dc-APGD operation in continuous-flow reaction-discharge systems, the synthesis of nanostructures is highly effective and possible to upscale and automate.

Due to the advantages of dc-APGD, in this paper the authors proposed the reaction discharge system for the unique flow-through synthesis of Re-based nanostructures that were found to be effective in catalytical processes. In these NTAP-based reaction-discharge systems, the solution containing Re(vii) anions acted as a flowing liquid anode (FLA) or flowing liquid cathode (FLC), depending on the polarity of a pin type counter electrode. The dc-APGD, generated between the above-mentioned two electrodes, leading to the effective production of ReNPs with Re^0^ and the Re oxide NPs depending on whether the precursor solution was the FLA or the FLC, respectively. The resulting nanomaterials were characterized using several experimental techniques in order to determine their optical and granulometric properties. Additionally, the plasma–liquid interactions responsible for the synthesis of the Re-based NPs were revealed. Afterwards, the ReNPs, synthesized with the aid of NTAP, were used as new nanocatalysts (NCats) for the model reaction of 4-nitrophenol (4-NP) hydrogenation.

## 2. Materials and methods

### 2.1. Reagents and solutions

All of the reagents were of an analytical grade or better. The de-ionized water was used in all the experiments. The raw-Re nanostructures were obtained from the precursor solution of ammonium perrhenate(vii) (NH_4_ReO_4_), which contains 2000 mg L^−1^ of Re(vii) ions. The reagents for the determination of the selected RNS and ROS were purchased from Hanna Instruments (Salaj, Romania) and Sigma-Aldrich (Steinheim, Germany), respectively. The reagents used for ROS determination were: (i) potassium iodide, (ii) starch, and (iii) agar, and they were purchased from Avantor Performance Materials (Gliwice, Poland), Sigma-Aldrich (Steinheim, Germany), and BTL (Lodz, Poland), respectively. The 4-NP and NaBH_4_ (MERCK, Branch Poland) used for the catalytic hydrogenation were applied as received.

### 2.2. The non-thermal atmospheric pressure plasma-based reaction-discharge system used for the production of raw-rhenium-based nanostructures

**Fig. 1:**
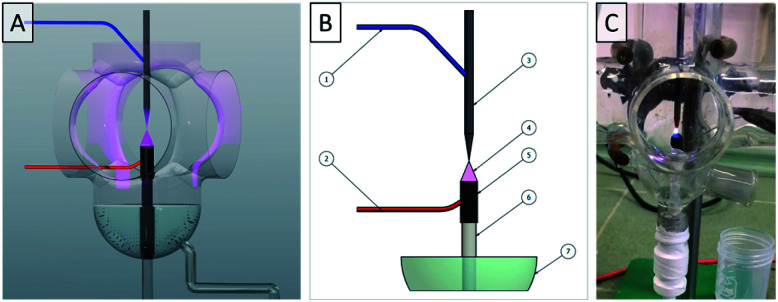
(A) Flow-mode reaction-discharge system. (B) Design of the FLC-dc-APGD and FLA-dc-APGD used for the production of raw-Re-based nanostructures: (1,2) high voltage (HV) inputs (HV+/HV- depending on the polarization: FLC or FLA); (3) tungsten electrode; (4) dc-APGD; (5) graphite tube; (6) quartz capillary; (7) sample with Re nanostructures. (C) Photograph of dc-APGD during operation.

The fabrication of raw-Re-based NPs was carried out in two dc-APGD-based reaction-discharge systems that were previously developed in our research group^10,15^ (Fig. 1) and working in the open-to-air atmosphere. To obtain a colloidal suspension of raw-Re-based nanostructures, the precursor solution was continuously introduced to a quartz discharge chamber by using a four channel peristaltic pump (Masterflex L/S, Cole-Palmer, Vernon Hill, USA) with a flow rate of 4.5 mL min^−1^. The Re-based NPs precursor solution was delivered to the quartz chamber by a quartz-graphite tube (OD = 4.00 mm/OD = 6.0 mm). Vertically to the quartz-graphite tube, there was a sharpened tungsten electrode mounted (OD = 3.2 mm) about 4 mm above the quartz-graphite tube. To provide a high-voltage of 1400 V to the electrodes, a dc-HV power supply provided by Dora Electronics Equipment (Wroclaw, Poland) was applied. The discharge current of 45 mA was maintained throughout and stabilized by applying a proper ballast resistor (including in the circuit). The power consumption during plasma operation was estimated to be 63 Watt. Additionally, electrical contact to the FLA or the FLA was provided by applying a Pt wire, which was connected to the graphite tube. The dc-APGD was operated in two configurations. When the Re(vii) solution acted as FLA (FLA-dc-APGD), the tungsten pin-type metallic electrode acted as a cathode. When the Re(vii) solution acted as a FLC (FLC-dc-APGD), the tungsten pin-type metallic electrode acted as an anode. Operating the dc-APGD for 1 h lead us to obtain about 270 mL of colloidal suspension, containing Re nanostructures. The resulting colloidal suspension of raw-Re-based nanostructures was gathered into 5.0 mL quartz vials. The samples, labelled as Re#FLA and Re#FLC, were then gathered for further analyses with regards to their optical, granulometric, and catalytic properties.

### 2.3. Characterization of the resulting nanomaterials

The optical properties of the raw-Re-based nanostructures produced with the aid of FLC-dc-APGD or FLA-dc-APGD were assessed using UV/Vis absorption spectrophotometry (Specord 210 Plus, Analytik Jena, Jena AG, Germany). The UV/Vis absorption spectra were acquired in the range from 200 to 900 nm with a resolution of 0.2 nm. The average size distribution of the Re-based nanostructures was assessed using the dynamic light scattering technique (DLS, Litesizer 500, Anton Paar GmbH, Austria). The measurements were performed in 3 mL polypropylene vials, and the collected data were processed using Kalliope software. The granulometric properties of the resulting Re-based nanostructures were estimated using transmission electron microscopy (TEM, Tecnai G^2^ 20 X-TWIN, ThermoFisher, MA, USA), which was supported by energy-dispersive X-ray spectroscopy (EDX) and selected area electron diffraction (SAED). The TEM measurements, followed by the EDX and SAED analyses, were carried out by placing one drop of the analysed solution onto a Cu–C grid. The solution was then left to dry out in an ambient atmosphere. The TEM-based average size distribution (by number) of the Re-based nanostructures was determined by determining diameter of each analysed nanoparticle with the aid of ImageJ software by counting at least 50 nanostructures per one photomicrograph.

### 2.4. Estimation of the plasma–liquid interactions

To reveal the processes occurring in the liquid phases at the plasma–liquid interface, quantitative analysis of the selected RONS was performed. Accordingly, the concentration of the NO_2_^−^ ions and the total concentrations of ROS (such as O), in addition to pH, were determined before and after the FLA-dc-APGD or FLC-dc-APGD treatment of the Re nanostructures' precursor solution.

To assess the concentration of NO_2_^−^, commercial available kits, provided by the Hanna Instruments (HI 96708, Salaj, Romania), were used. The analysis was carried out following the manufacturer's instructions. The 2000 mg L^−1^ solution of Re(vii) ions, was introduced to the FLC-dc-APGD or FLA-dc-APGD reaction-discharge system during its stable operation (see Fig. 1 for more details). The raw-Re-based nanostructures were instaniously produced and their colloidal suspensions (5.0 mL) were collected into separate glass vials and then diluted with de-ionized water to 10.0 mL. To determine the concentration of the NO_2_^−^ ions, the kit reagents were mixed with the diluted colloidal suspension of the raw-Re-based nanostructures according to the manufacturer's instructions and the product of the colour-forming reaction was measured. In the same manner, the concentration of NO_2_^−^ in the untreated 2000 mg L^−1^ of Re(vii) ions was examined. The standard deviation (SD) values for the concentration of NO_2_^−^ were assessed based on three independent experiments for each polarization of the discharge system (using Prism 8.0 Graphpad Software) (San Diego, CA, USA).

In turn, the concentration of the total ROS was estimated using the KI-starch-based method.^33^ In this assay, the de-oxidative potential of the ROS, such as OH˙, O, O_3_, H_2_O_2_, and HO_2_^−^, was established. The colour-forming solution was prepared by heating the reaction-mixture, *i.e.*, 0.3% (m/v) of potassium iodide and 0.5% (m/v) of starch, up to 70 °C. The colloidal suspension, containing raw-Re-based nanostructures was diluted three times and 2.90 mL of the so-prepared solution was mixed with 0.10 mL of the chilled KI-starch reagent in the 24 well-test plate. After 30 min the progression of the reaction was observed and the resultant mixture was transferred into a quartz cuvette to measure the absorbance values at 590 nm using Specord 210 Plus instrument (Analytik Jena, Jena AG, Germany). The concentration of the total ROS was calculated based on a calibration curve, which was prepared using 5 points of the standard test performed using the KI-starch reagent and a H_2_O_2_ solution.

For a better understanding of the differences between the oxidative potential of the FLC-dc-APGD system and the FLA-dc-APGD, the spatial distribution of the total ROS was investigated as follows. The proper amounts of the 0.3% (m/v) KI, 0.5% (m/v) starch, and 0.9% bacteriological agar solutions were mixed and heated nearly to boiling point. Next, the resulting homogenous mixture was poured out into a standard Petri dish, with a gel plate being obtained (*ca.* 8.00 mm thick). The so-prepared plate was divided into smaller round-shape samples (diameter ∼14.00 mm). Then, they were put on top of the FLA or the FLC, after which FLC-dc-APGD or FLA-dc-APGD were ignited. The occurrence of the discharge on the gel surface led to the appearance of a blue circle-like region, where a darker shade indicated significant ROS generation. Afterwards, the interaction between the NTAP source and the resulting gel surface was captured in 3 phases: Phase I – the ignition of the NTAP, Phase II – the contact of the Re-based NPs precursor solution with the NTAP during the synthesis, Phase III – the extended treatment of the NTAP in reaction-discharge system.

Finally, to reveal how the FLC-dc-APGD and FLA-dc-APGD affected the pH of the Re-based NPs precursor solution subjected to the NTAP treatment, the pH of untreated as well as dc-APGD-treated solution was measured with the aid of a CPC-505 pH meter (Elmetron, Zabrze, Poland).

### 2.5. Evaluation of the catalytic activity

The uncoated Re-based nanostructures, obtained using FLA-dc-APGD or FLC-dc-APGD, were used as homogenous NCats for the catalytic hydrogenation of 4-NP to 4-aminophenol (4-AP). The model reaction was carried out in the following way. First, 2.7 mL of 4-NP (0.1 mmol L^−1^) and 0.1 mL of NaBH_4_ (0.1 mol L^−1^) solutions were introduced into a 3 mL quartz cuvette. Then, 0.05 mL of the suspension solution containing the prepared Re-based NPs (ReNPs or Re oxide NPs) was introduced, after which the hydrogenation began. The data for the evaluation of catalytic activity were collected using UV/Vis absorption spectrophotometry (JASCO V-570, MD, USA) by monitoring the changes of absorbance at the wavelength of 400 nm. The band observed at this wavelength is assigned to the 4-nitrophenolate anion (O_2_N–Ar–O^−^), which is gradually reduced in the process.

The collected data were then re-calculated using the pseudo-first order kinetic model. The calculations were made with the assumption that the change of absorbance is proportional to the change of 4-NP concentration. This enabled the drawing of ln *A*_*t*_/*A*_0_*vs. t* plots (*A*_*t*_: absorbance at time *t*, *A*_0_: initial absorbance at 400 nm). Then, the first-order kinetic model's apparent rate constant (*k*_app_) was taken from the slope of the above-mentioned curves. Finally, the time-dependent conversion (%) of 4-NP was re-calculated to turnover frequencies (TOFs) in order to enable the molar activity of the NCats to be assessed. This was done using the following eqn (1):^34^

1 TOF = *n*_4-NP_*rn*_Re_^−1^*t*^−1^

where *n*_4-NP_ and *n*_Re_ are the moles of the 4-NP and Re-based NPs, respectively, *t* is the process time (min), and *r* is the yield (%) of reduction.

## 3. Results and discussion

### 3.1. Optical and structural properties of rhenium-based nanomaterials

**Fig. 2:**
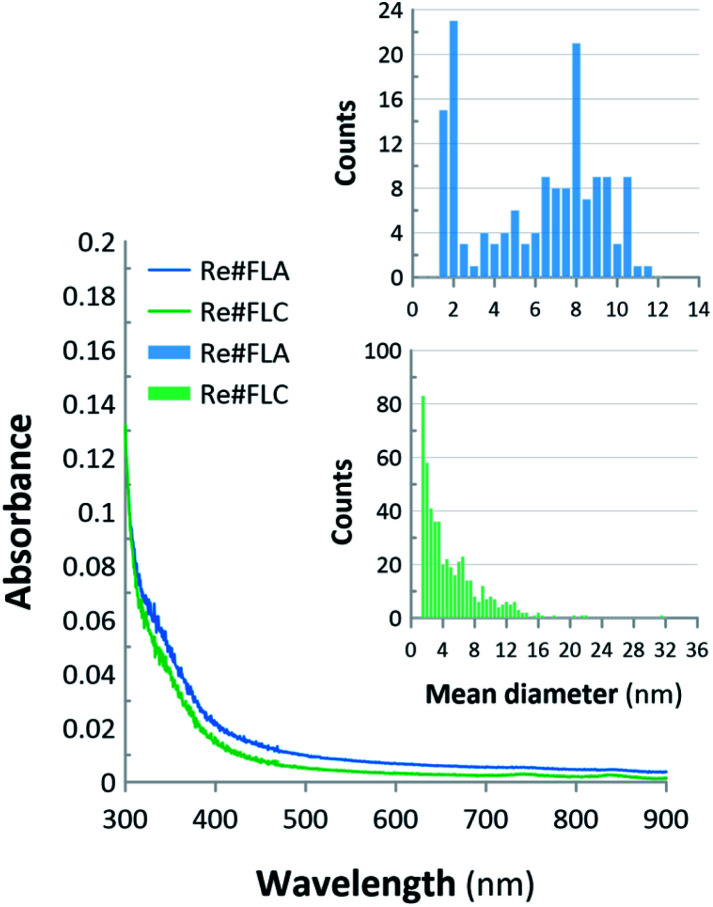
UV/Vis absorption spectra and the average size distribution (by number) of the Re-based nanostructures. The average size distribution of the analysed nanostructures was calculated based on the TEM photomicrographs.

**Fig. 3:**
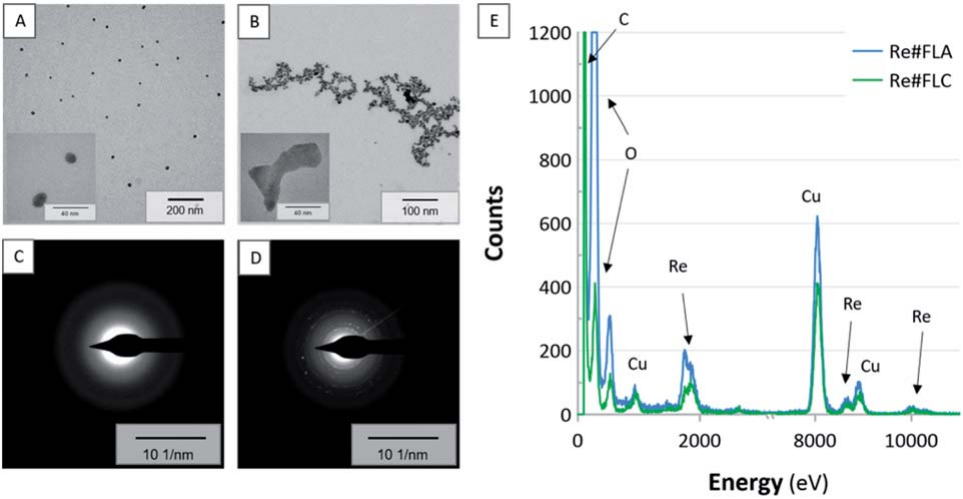
The representative TEM photomicrographs, SAED patterns, and EDX spectra of the Re#FLA (A, C, E) and Re#FLC (B, D, E).

Firstly, the optical properties of the produced Re-based nanostructures were assessed using UV/Vis absorption spectrophotometry. Secondly, the structural properties of these nanostructures obtained with the aid of dc-APGD (the Re#FLA material and the Re#FLC material) were assessed using TEM supported by EDX and SAED in order to reveal their morphology, elemental composition, and crystalline structure. In Fig. 2, the acquired UV/Vis absorption spectra are correlated with the nanostructures' size distribution. This size distribution was calculated based on the TEM photomicrographs (Fig. 3A and B), where the nanostructures with visible grain boundaries are displayed.

Generally, Re-based nanostructures produced *via* plasma-liquid interactions in the flow-through reaction-discharge system based on dc-APGD revealed a very small dielectric constant value. Therefore, they did not show any characteristic maxima in the UV/Vis absorption spectra. As can be seen in Fig. 2, no band at ∼240 nm, appearing due to the transition from the O^2−^ ion to the Re^7+^ ion at the centre of the ReO_4_^−^ oxoanion complex, was either detected.^27^ Therefore, as reported in ref. 27, it was concluded that all of the Re(vii) ions were reduced in the solution due to the interactions of these ions with dc-APGD components, *i.e.*, the RNS, the ROS, and the e_aq_^−^.

The TEM photomicrographs show significant differences in the morphology of the resultant nanostructures, *i.e.*, the Re#FLA material and the Re#FLC material (see Fig. 3A and B, respectively). In the case of Re#FLA, the synthesized NPs were monodisperse, spherical in shape, and had an average size distribution of 6.02 ± 3.01 nm (Fig. 3A). The Re-based nanostructures in the Re#FLC had the entirely different morphology. In this case, the bone-like and cubic-like NPs were detected, which were characterized by an average size of 4.97 ± 3.82 nm (Fig. 3B). Additionally, some aggregates for these NPs were noted.

To determine the crystalline structure of the analysed Re#FLA and Re#FLC materials, the SAED technique, performed during the TEM measurements, was employed. As can be seen in Fig. 3C and D, corresponding to the Re#FLA and Re#FLC, respectively, different SAED patterns were assessed. The SAED pattern corresponding to the Re#FLA (Fig. 3C) is blurred, which suggests that the analysed Re-based NPs were mainly amorphous in their structure. Simultaneously, the EDX spectrum (Fig. 3E) confirms that the investigated NPs indeed contained the Re-based nanostructures. It can therefore be concluded that the Re#FLA material probably contained NPs based on the Re–Re bonds, the characteristic feature that shifts the SAED pattern to the amorphous region.^35–37^ This in turn might suggest a major content of Re^0^ in the form of ReNPs, and such an effect is usually observed when there is no oxide- or sulphide doping.^35–37^

Considering the SAED pattern acquired for the Re#FLC (Fig. 3D), it was quite different when compared to the Re#FLA sample. In this case, the determined *d*-spacings were calculated as follows: 2.87, 3.70, and 3.80 Å. This might correspond to the fingerprints of the ReO_2_NPs, ReNPs (oxide- or sulphide-doped), and ReO_3_NPs, respectively.^23,38,39^ Furthermore, the calculated d-spacing values corresponded to the (100), and (101) lattice fingers of the ReO_3_-cubic crystals,^39^ proving that such NPs were indeed present in the Re#FLC sample.

To reveal the elemental composition of the obtained Re-based nanomaterials, the EDX analyses were conducted during the TEM measurements as well. As can be seen in Fig. 3E, Re was found in both the analysed materials *i.e.* the Re#FLA and Re#FLC. Additionally, Cu (from the Cu grid), C (from the sample holder), and O (from the oxide-doped Re-based NPs) were detected.

Based on all the results, it was concluded that FLA-dc-APGD led to the synthesis of ReNPs (Re#FLA), while FLC-dc-APGD led to the synthesis of oxide-doped Re nanostructures (Re#FLC). This difference in the type of the products of the dc-APGD synthesis could be explained by the difference in the plasma-liquid interactions occurred in both applied NTAP systems, *i.e.*, FLA-dc-APGD and FLC-dc-APGD, which are described in Section 3.2.

### 3.2. Plasma–liquid interactions leading to the synthesis of monodisperse Re-based nanostructures

**Fig. 4:**
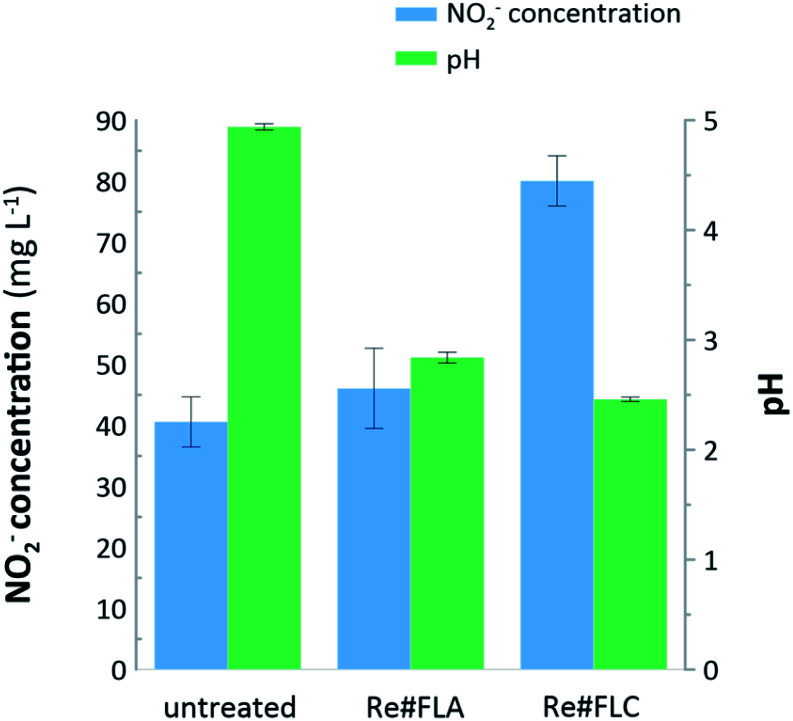
Concentration of NO_2_^−^ ions and the pH determined in the untreated, FLC-dc-APGD-treated, and FLA-dc-APGD-treated Re-based nanostructures precursor solutions.

To reveal the mechanisms occurring at the plasma–liquid interface, the concentration of selected RONS was monitored. First, the content of NO_2_^−^ ions was determined. Based on the results displayed in Fig. 4, it can be stated that the concentration of RNS (80.06 ± 4.10 mg L^−1^) must have contributed to the synthesis of the Re#FLC. Interestingly, the concentration of NO_2_^−^ ions in the untreated NH_4_ReO_4_ solution and Re#FLA remained close to each other (40.58 ± 4.10 mg L^−1^ and 46.06 ± 6.58 mg L^−1^, respectively), indicating that no RNS participated in the fabrication of the Re#FLA. The production of the NO_2_^−^ ions could also be roughly correlated with changes in the measured pH values. The pH of the untreated precursor solution was 4.94 ± 0.03, while the pH of the suspensions containing the Re#FLA and Re#FLC was 2.84 ± 0.05, and 2.46 ± 0.02, respectively. The solution containing the Re#FLC noted the greatest pH decrease, which was possibly associated with the contribution of the NO_2_^−^ ions in the synthesis of the Re oxide nanostructures. The support for this conclusion can be found in the production of HNO_3_, which can be synthesized according to the reaction: NO_2_ + OH = HNO_3_.^15^ The decrease of pH can also be caused by the increased concentration of H_3_O^+^ ions at the plasma-liquid interface.^15^

Based on the quantitative assessment of the oxidative potential of the suspensions containing raw-Re-based nanostructures right after their synthesis, a similar situation was observed in the case of the total ROS concentration. The results indicate that the oxidative potential of the untreated NH_4_ReO_4_ solution was near 0. When the suspension contained the Re#FLC, the total ROS concentration was 48.94 ± 1.04 mg L^−1^, while in the case of the suspension of the Re#FLA sample it was 34.27 ± 1.44 mg L^−1^.

To sum up, the synthesis of Re#FLC was associated with the more abundant concentrations of RNS and ROS (Fig. 4), as well as the most significant drop of pH as compared to these observed for the synthesis of Re#FLA. This could be related to the dissimilar operating mechanism in the FLC-dc-APGD and FLA-dc-APGD. In the case of the dc-APGD system with the FLC solution (Re#FLC), the surface of the Re(vii) solution was exposed to the bombardments by the positive ions, *i.e.*, H_2_O^+^.^12,13,40^ In this case, the high-energy H_2_O^+^ ions could ionize water molecules, leading to the formation of the e_aq_^−^ (H_2_O^+^ + H_2_O = H_2_O^+^ + e_aq_) that were likely responsible for the reduction of the ReO_4_^−^ ions. Nevertheless, the low-energy H_2_O^+^ ions could recombine with the water molecules, leading to the generation of the hydroxide radicals (OH˙) and the hydrogen peroxide molecules (H_2_O_2_) in the following reactions: H_2_O^+^ + H_2_O = H_3_O^+^ + OH˙, OH˙ + OH˙ = H_2_O_2_.^12,13,40^ In turn, the formation of Re oxide nanostructures could be possible in the case of the Re#FLC sample. Conversely, when the surface of the Re(vii) solution was bombarded by the high-energy electrons, as was the case of the FLA-dc-APGD system, a high amount of the e_aq_ was available for different reactions in the solution, including the direct reduction of the ReO_4_^−^ ions to the Re^0^ form, and the recombination with the H_3_O^+^ ions to produce the H radicals (H˙), also having a high reductive potential.^12,13,40^ This difference in the operating mechanism of FLC-dc-APGD and FLA-dc-APGD could be the reason why the monodisperse bone-like and cubic-like morphology NPs found in the Re#FLC were recognized as Re oxide nanostructures, while the NPs found in the Re#FLA were recognized as ReNPs.

**Fig. 5:**
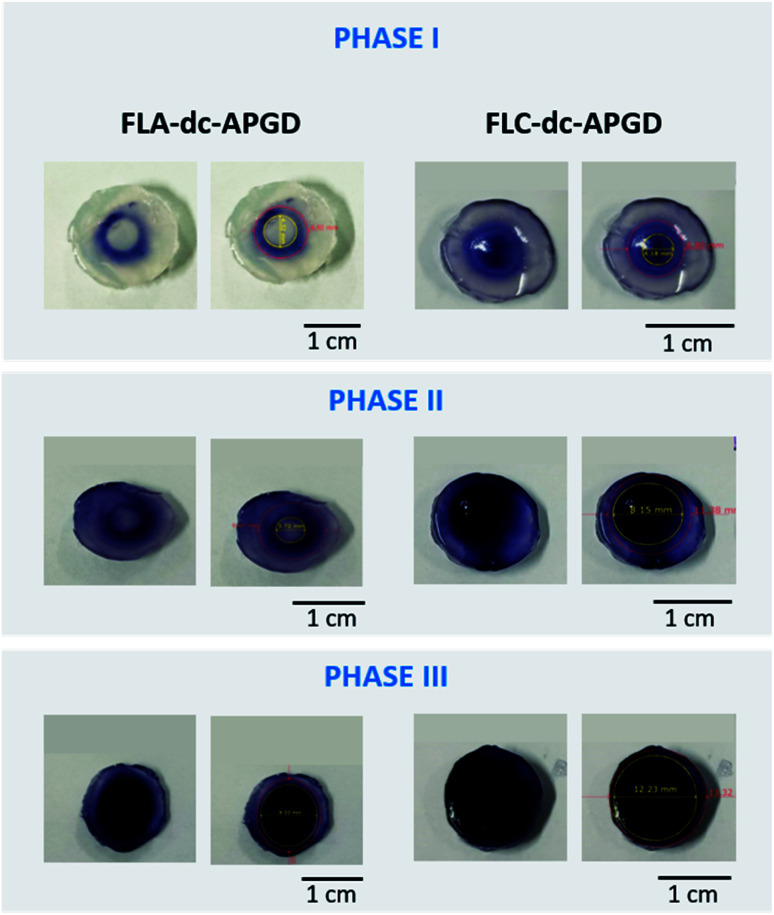
The spatial distribution of the generated total ROS during the irradiation of the KI-starch gels' surface with dc-APGD operated in the FLA or FLC configuration. Phase I – the moment of the dc-APGD ignition on a droplet of the ReO_4_^−^ solution – estimated to be a time of less than 1 s, Phase II – the synthesis of the Re-based nanostructures in the dc-APGD reaction discharge system (the average time of ∼2 s), Phase III – prolonged irradiation contact with the surface on a droplet of the precursor solution (∼4 s).

To support for the above-mentioned hypothesis about the production of the Re-based NPs *via* the plasma–liquid interactions, the spatial distribution of the generated ROS on the surface of the prepared solid KI-starch gels in the case of the FLA-dc-APGD and the FLC-dc-APGD systems was determined (Fig. 5). During the Phase I (discharge ignition), which was roughly estimated to be within the 1^st^ s of the contact of the plasma with the droplets of the precursor solution, the appearance of a ring-shape colorization was observed. In the case of the FLA-dc-APGD configuration (Re#FLA), the untreated area was equal to 4.32 mm, and the oxidative area was equal to 6.92 mm. The situation is different in the case of the FLC-dc-APGD configuration (Re#FLC), which displays a negligible untreated area and a ROS generation circle area with a diameter of 4.18 mm. The circle is further extended to the oxidized area, which has a diameter of 6.80 mm.

Further differences were observed in Phase II of the dc-APGD operation. In the case of the FLA-dc-APGD configuration, there was a lightly coloured area with a diameter of 3.72 mm, and a darker region with a diameter of 9.67 mm. However, in the case of the FLC-dc-APGD configuration, there was deeply dark zone with a diameter of 8.15 mm, and also a light blue sector (diameter of 11.38 mm). Finally, during the Phase III of the prolonged irradiation of the KI-starch gels' surface with the dc-APGD system, further changes were observed. In the case of the Re#FLA, it can be observed that the central irradiated region with a diameter of 8.33 mm turned into a deep blue colour, while the diameter of the total reacted area was 10.80 mm. In the case of the FLC, a much larger, deeply blue coloured region with a diameter of 12.23 mm was observed, while the totally colorized region was 13.32 mm.

These results strongly correlate with the total ROS concentration determined in the post-treated suspensions containing the Re-based nanostructures, and the hypothesis of a more oxidative environment in the case of the FLC-dc-APGD system, due to the possible production of the OH˙ and H_2_O_2_ species. Indeed, as was shown in the above-described experiment, the total ROS concentration was significantly elevated when the FLC configuration was used. A high oxidative potential observed for this polarization could be the reason for the synthesis of the ReO_2_ and ReO_3_ nanostructures in addition to the metallic Re nanostructures.

### 3.3. Catalytic hydrogenation of 4-NP

**Fig. 6:**
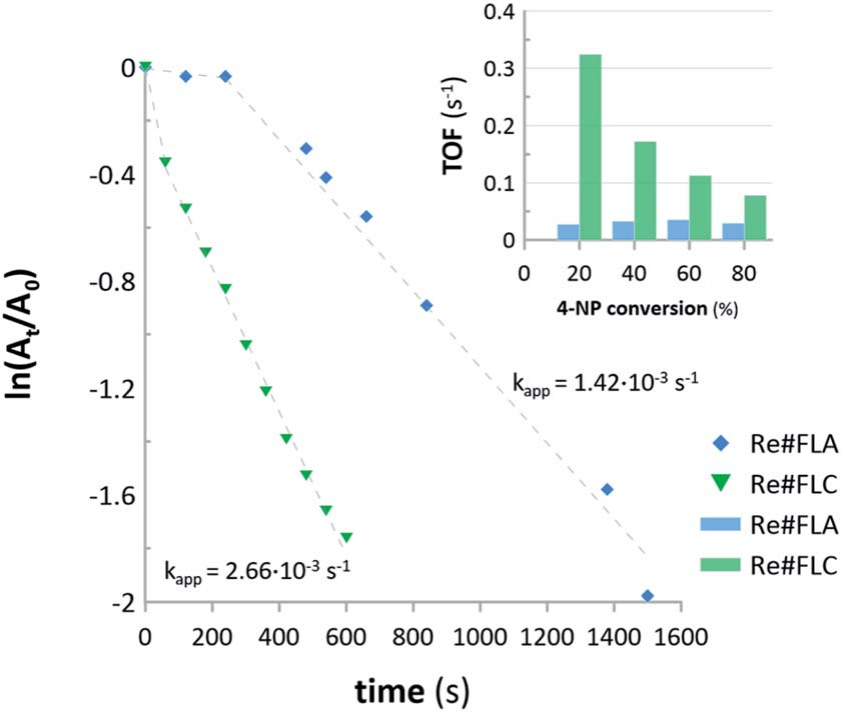
First-order kinetic plots and TOF values for the hydrogenation of 4-NP with the aid of Re#FLA and Re#FLC samples used as the homogenous NCats.

The Re#FLA and Re#FLC samples, containing ReNPs and Re oxide NPs, respectively, were used as homogenous catalysts for the hydrogenation of 4-NP. The reactions were monitored by registering the absorbance at 400 nm (attributed to the reduced 4-nitrophenolate anion). The collected data were then re-calculated using pseudo-first order kinetics in order to draw the corresponding plots, which are displayed in Fig. 6.

Based on the results, it can be concluded that both NCats led to a 85% conversion of 4-NP. However, there is almost a two-fold difference in the apparent rate constants (*k*_app_) between the two synthesized Re-based nanomaterials. The Re#FLC NCat led to a 85% 4-NP reduction within 10 min, with *k*_app_ = 2.66 × 10^−3^ s^−1^, while the Re#FLA NCat achieved the same conversion of 4-NP within 25 min, with *k*_app_ = 1.42 × 10^−3^ s^−1^. This effect could be attributed to the difference in the average size of the NPs found in the Re#FLA and Re#FLC samples. Although the difference in the average size of both Re-based NPs (∼6 nm for the ReNPs *versus* ∼5 nm for the Re oxide NPs) seemed to be too small to make such a significant impact on the catalytic activities of both NCats, there could be a link, however, between the achieved catalytic effectiveness and the average size distribution of these NPs (see Fig. 2 for details). Based on these results, it can be stated that the Re#FLC sample is represented by a significantly greater number of smaller NPs that do not exceed the size of 2–4 nm. Simultaneously, the Re#FLA samples mostly contained the NPs of 8–10 nm in size. The same histograms (Fig. 2) revealed yet another difference: the Re#FLA seems to be more dispersed (based on the count number). Moreover, as evidenced by the calculated TOFs (see Fig. 6), in the initial stage of the reaction (20% of 4-NP conversion), the TOF value of the Re#FLC was 20-fold greater when compared to the TOF of the Re#FLA NCat. This tendency remained, although the TOFs in the case of the Re#FLC decreased as the reaction advanced. All of the observations mentioned above allow for to come to the conclusion that the Re#FLC provided a more active surface of the Re-based NPs, in turn leading to the more efficient reduction of 4-NP. It must also be noted that the Re#FLA sample (recognized as ReNPs) clearly revealed a 400 s induction period before the actual hydrogenation of the 4-NP (Fig. 6). The explanation for this phenomenon was found in the acquired UV-Vis spectra of the reaction mixture (results not shown here). In this initial period, the gradual decrease of the absorbance at 400 nm (attributed to the hydrogenation of 4-NP) did not entail the appearance of the band at 295 nm attributed to the presence of 4-AP. At the reaction time of >400 s, the aforementioned band at the *λ*_max_ (295 nm) suddenly appeared and rapidly increased with time. This suggests that 4-NP was adsorbed on the surface of the Re#FLA NCat. However, the product of the catalytic reduction, *i.e.*, 4-AP, was possibly held on the surface of the Re#FLA NCat at the reaction time of <400 s and was released at higher reaction times.

Re-based NPs catalysts were not commonly referred to in scientific literature. This has resulted in a lack of reports that provide insights into Re-based homogenous catalysts for the hydrogenation of nitroaromatic compounds. Based on the literature review, there are two examples where ReNPs were used as NCats for the reduction of nitroarenes. Therefore, the present results were also compared to other works related to the reduction of nitroarenes (see Table 1). The Re#FLC NCat obtained in this work provides an advancement when related to ReNPs encapsulated in poly(allylamine hydrochloride) (*k*_app_ = 2.53 × 10^−3^*vs.* 2.66 × 10^−3^ s^−1^).^27^ Certainly, the literature also provides much more efficient Re-based homogenous catalysts based on a carbon-nanotube support that significantly boosts the contact surface between nitroaromatics and ReNPs.^23^ However, the methodology proposed in the both cited works was quite complex and required the additional supports and reducing agents for the production and the stabilization of Re-based nanostructures. Hence, it can be concluded that the Re-based nanomaterials produced in the present work could be obtained in a much simpler and faster way. The added value of the proposed plasma synthesis was the possibility of obtaining an unlimited amount of both NCats and the possibility of rescaling the reaction-discharge system based on dc-APGD or its automation.

**Table 1:** Comparison of recent Re-based homogenous catalysts for the reduction of nitroaromatic compounds

Catalyst	Method for synthesis	NAR^a^	*D*_H_ b [nm]	*k*_app_ c × 10^−3^ [s^−1^]	Ref.
ReNPs/PAH	Reduction of Re^7+^ over poly(allylamine hydrochloride) scaffold	4-NA	1.7 ± 0.3	2.53	27
			0.7 ± 0.25	1.26	
ReNPs/OMC	Solvent-evaporation induced self-assembly of Re_2_(CO)_10_ on ordered mesoporous carbon nanotubes	4-NP	5.0 ± 0.2	149.8	23
		4-NA		147.2	
		2-NA		145.4	
		2,4-DNP		146.9	
		2,4,6-TNP		144.2	
ReNPs	Reduction of Re^7+^ in a reaction discharge system, with FLA-dc-APGD as the source of NTAPs	4-NP	6.0 ± 3.0	1.42	This work
Re oxide NPs	Reduction of Re^7+^ in a reaction discharge system, with FLC-dc-APGD as the source of NTAPs		4.97 ± 3.8	2.66	This work

a NAR: nitroaromatic compound; 4-NP: 4-nitrophenol; 4-NA: 4-nitroaniline; 2-NA: 2-nitroaniline; 2,4-DNP: 2,4-dinitrophenol; 2,4,6-TNP: 2,4,6-trinitrophenol.

b Average size of NPs.

c Apparent rate constant (pseudo-first order).

## 4. Conclusions

In the present work we have presented a fast, simple, and effective for the synthesis of Re-based nanostructures with defined optical and structural properties, in the view of their application in the catalytic hydrogenation of nitroaromatic compounds. The method of synthesis involves the application of highly-throughput NTAP-based reaction-discharge systems, where dc-APGD serves as a source of RNS and ROS, which are able to reduce the ReO_4_^−^ ions and electrostatically stabilize the resultant Re-based nanostructures in the solution. To achieve an efficient production of Re-based nanomaterials, two configurations of the reaction discharge system were used. The first system involved the application of the ReO_4_^−^ ions solution as the FLA, the second one as the FLC. As compared to the FLA-dc-APGD configuration, the latter (FLC-dc-APGD) configuration resulted in the formation of the excessive RNS and ROS, which contributed to the reduction of Re(vii) ions and their complete or partial oxidation. As a result, the bone-like and cubic-like Re oxide NPs with an average size of 5.0 ± 3.8 nm were produced. In the case of the FLA-dc-APGD system, the spherical ReNPs with Re^0^, having an average size of 6.0 ± 3.0 nm, were produced.

Both types of nanomaterials can be easily used as homogenous catalysts for the hydrogenation of 4-NP to 4-AP. 2, 3 all this makes that the Re-based nanomaterials can be considered as really prospective NCats, and the use of non-thermal plasmas for producing thereof is definitely worth further investigations.

## Author contributions

P. C. and A. D. conceptualized this work and wrote the first draft of the manuscript; the manuscript was corrected with contributions from all the authors. A. D. and P. C. prepared the answers to Reviewers' questions and comments. All the authors have given approval to the final version of the manuscript A. D. and P. J. employed the method for the synthesis of the Re nanostructures; P. J. conducted the UV/Vis absorption analyses and DLS measurements; P. C. performed TEM, SAED, and EDX analyses, and the research on catalytic activity; P. C. and A. D. evaluated the granulometric and optical properties of the Re nanostructures; A. D. and D. T. performed the determination of the RNS and ROS concentrations, and participated in the discussion of the results. P. P. corrected the manuscript and participated in the discussion of results related to the mechanisms occurring at the plasma–liquid interface.

## Conflicts of interest

There are no conflicts to declare.

## Supplementary Material
